# Association between Obesity and Overweight and Cardiorespiratory and Muscle Performance in Adolescents

**DOI:** 10.3390/ijerph18010134

**Published:** 2020-12-27

**Authors:** Peter Petrovics, Barbara Sandor, Anita Palfi, Zsolt Szekeres, Tamas Atlasz, Kalman Toth, Eszter Szabados

**Affiliations:** 1 1st Department of Medicine, Division of Preventive Cardiology and Rehabilitation, University of Pecs, Medical School, H-7623 Pecs, Hungary; pepeti01@gmail.com (P.P.); sandor.barbara@pte.hu (B.S.); palfi.anita@pte.hu (A.P.); szekeres.zsolt@pte.hu (Z.S.); 2 Institute of Physical Education and Sport Sciences, Faculty of Sciences, University of Pecs, H-7624 Pecs, Hungary; attam@gamma.ttk.pte.hu; 3 1st Department of Medicine, Division of Cardiology and Angiology, University of Pecs, Medical School, H-7624 Pecs, Hungary; toth.kalman@pte.hu

**Keywords:** cardiorespiratory performance, muscle performance, adolescents, obesity, overweight

## Abstract

The high prevalence of obesity in childhood and adolescence has major public health consequences, since it is associated with various chronic diseases in the short- and long-term. The goal of our study was to examine the possible association between obesity and overweight and cardiorespiratory and muscle performance during a 4-year follow up period in adolescents. The body mass index (BMI) and physical performance of adolescents (360 girls and 348 boys) between 14–18 years of age was measured twice a year, and the possible correlation between overweight and obesity and cardiorespiratory and muscle performances were investigated. Our results revealed that cardiorespiratory performance increased significantly in boys during the 4 years (*p* < 0.001), but the aerobic performance of girls only showed seasonal fluctuation. Muscle performance significantly increased both in boys and girls (*p* < 0.001). Inverse association between obesity and cardiorespiratory and muscle performance was proved. Overweight was also inversely correlated with cardiorespiratory performance, but it demonstrated no correlation with muscle strength. Avoiding increased BMI and decreased physical fitness is essential for adolescents’ health to prevent short- and long-term adverse effects.

## 1. Introduction

The worldwide prevalence of obesity among adults as well as in children and adolescents has markedly increased over the past three decades leading to the so-called obesity epidemic. According to the latest data in the literature, childhood and adolescent obesity is as high as 18.5% in the USA [1], and 15% in Europe [2]. The prevalence of obesity in adolescence has stabilized at a high level in developed countries [3,4,5] but is still increasing in developing countries [5,6,7]. Besides many short-term effects of obesity such as cardio-metabolic, respiratory, musculoskeletal, endocrine, psychosocial, increased cancer risk, etc. [8,9,10,11], a high percentage of children and adolescents track their obesity into adulthood [12] resulting in several chronic diseases [13] and even premature death [14].

It is recommended that children and adolescents aged 6–17 years do 60 min or more of physical activity each day [15]. Despite guidelines and recommendations [3,6,15,16], a decline in physical activity (PA) and cardiorespiratory and muscular fitness levels [17,18] has been reported worldwide among children and adolescents [19,20]. Cardiorespiratory fitness is defined as the overall capacity of the cardiovascular and respiratory systems to provide adequate amount of oxygen to the body during prolonged or strenuous exercise. Low cardiorespiratory fitness in children and adolescents has been associated with increased body fatness [20,21], hypertension, [20,22] increased risk of metabolic syndrome [9,18,23], and worse academic performance [24,25]. Besides cardiorespiratory fitness, muscular fitness is independently and inversely associated with clustered metabolic risk during adolescence [26]. The main health-related muscular fitness components are maximal (isometric and dynamic), explosive, endurance and isokinetic strength [25]. Muscular endurance is the ability of a muscle or muscle group to perform repeated contractions. A large meta-analysis revealed negative association between muscular fitness in childhood and adolescence and adiposity and cardiometabolic parameters in adulthood. The effects of endurance (push-ups, sit-ups, bent arm hang, etc.) and strength tests (handgrip, standing long jump, vertical jump, etc.) were similar. [27]. Furthermore, a negative association between standing long jump test, which assesses lower-limb maximal dynamic contraction, and total cholesterol in overweight and obese male adolescents was observed [28].

Previous studies have shown a strong correlation between increased body mass index (BMI) and reduced cardiorespiratory fitness in children and adolescents [4,29]. The association between increased body weight and decreased cardiorespiratory fitness is unequivocal in most of the studies [30,31,32] but its interaction with muscle performance is more ambiguous [9,26]. Furthermore, most of the studies examining the association between childhood and adolescence physical fitness and BMI are cross-sectional. In our study, we have measured the cardiorespiratory and muscle performance of adolescents between 14 and 18 years of age twice a year and investigated the possible association between overweight and obesity and cardiorespiratory and muscle performance and observed age and gender differences. Our aim was to investigate whether overweight shows the same relationship to cardiorespiratory and muscle performance as obesity.

## 2. Materials and Methods

### 2.1. Subjects

A total of 708 students from four-grade high school classes were enrolled in the study (360 girls with an average age of 14.2 ± 0.4 years, and 348 boys with an average age of 14.1 ± 0.4 years) at the beginning of high school (9th grade) in Baranya county, Hungary. Measurements (body weight and height, cardiorespiratory and muscle performance) were performed twice a year (autumn and spring) for 4 years, until the end of high school (12th grade). The inclusion criteria were as follows: students who had just started high school and agreed to participate in the study. Exclusion criteria were any medical conditions that prevented student attending physical exercise classes or did not agree to participate in the study. A written informed consent was obtained from the adolescents for the measurements and the anonymous use of data for scientific purposes. Parents were also asked to sign the form to allow the measurements and data handling. The study was approved by the Regional Ethics Committee of the University of Pecs.

### 2.2. Body Weight, BMI, and Obesity Measurements

Body weight was measured to the nearest 0.1 kg using an electronic digital body weight weighing scale, and height was measured to the nearest 0.1 cm with a manual height board. To screen for overweight and obesity, body mass index (BMI) and sex- and age-specific BMI-for-age were calculated using the BMI-for-age BMI growth charts [19]. Adolescents with a BMI-for-age ≥95th percentile were considered obese, between the 85th and 95th percentiles were classified as overweight, and with a BMI-for-age of <85th percentile were considered normal. The cut-off value for underweight was less than the 5th percentile of the BMI-for-age [7,19].

### 2.3. Measurements of Cardiorespiratory Performance

For the assessment of the cardiorespiratory performance of the adolescents, the 12-min run–walk test was used. The students ran, jogged, or walked on a flat course as far as they could in 12 min, and the distance covered was recorded in meters [33].

### 2.4. Measurements of Muscle Performance

Muscle performance was assessed using three motor tests. Standing long jump test: To assess leg dynamic muscle strength, standing long jump tests were performed. Children stood behind a line marked on the ground and attempted to jump as far as they could, landing on both feet without falling backwards. The measurement was taken by a tape measure from the take-off line to the nearest point of contact, with 1 cm accuracy, on the landing (back of the heels). A maximum of three attempts were allowed, and the best result was recorded in centimeters [34]. Push-up test: The endurance and dynamic strength of shoulder and arm muscles were measured by push-up tests. Students bent and stretched the arms in a push-up position with only the hands and the toes touching the floor, while their torso remained straight. They performed as many repetitions as they could until exhaustion, and the number of push-ups was recorded [35]. Sit-up test from supine position: To measure the endurance and dynamic strength of abdominal muscles, sit-up tests were performed. Students lied on a mat on their back while bending both knees at 90-degree angles, keeping their feet on the mat, pointing the elbows forwards, putting the fingers behind the ears, and flattening the stomach. They raised the torso off the floor, touching their thighs with the elbows, then descended back and returned to the starting position with quick steady tempo/space until exhaustion or for a maximum of 4 min. The test ended when the students were no longer able to continue the sit-ups or until the end of the 4th minute. The number of sit-ups was recorded [35].

### 2.5. Statistical Analysis

The significance level was defined as *p* < 0.05. IBM SPSS statistical software (New York, NY, USA), version 11.0.1 was used to conduct descriptive analyses and to describe the sample. According to the Kolmogorov–Smirnov normality test, data collection revealed a significant deviation from the normal distribution. Therefore, the nonparametric Friedman test together with the post-hoc analysis through Wilcoxon signed-rank tests were conducted with a Bonferroni correction to analyze potential changes between gender groups, and the nonparametric Kruskal–Wallis test was performed to describe potential changes between the different weight subgroups.

A gender specific sample size and power analysis was performed for the investigated population using PS program version 3.1.2. For the sample size of *n* = 254 boys needed to detect a true difference of δ = 10.25 in sit-up test values with 90.08% power, where type I error probability is α = 0.05. For the sample size of *n* = 255 girls needed to detect a true difference of δ = 8.28 in sit-up test values with 93.52% power, where type I error probability is α = 0.05.

## 3. Results from Gender within-Groups Analyses

### 3.1. Changes in the BMI-for-Age during the 4 Years

At the beginning of our study, 4 percent of the girls were underweight, 75 percent normal weight, 15 percent overweight, and 6 percent obese. With respect to the boys, 6 percent were underweight, 69 percent normal weight, 15 percent overweight, and 10 percent obese. In the spring of 12th grade, 8 percent of the girls were underweight, 75 percent normal weight, 12 percent overweight and 5 percent obese. With respect to the boys, 7 percent were underweight, 72 percent normal weight, 11 percent overweight, and 10 percent obese. Among girls, the average baseline BMI-for-age of 20.52 ± 2.87 did not increase significantly during the 4-year observational period, as it was 21.01 ± 3.05 at the end of the 12th grade (*p* = 0.120). Similarly, there was no significant change in the average BMI-for-age of the boys. It was 20.80 ± 3.75 in the autumn of 9th grade, and 22.01 ± 3.71 in the spring of 12th grade (*p* = 0.100).

#### 3.1.1. Cardiorespiratory Performance of Boys and Girls during the 4 Years

**Figure 1:**
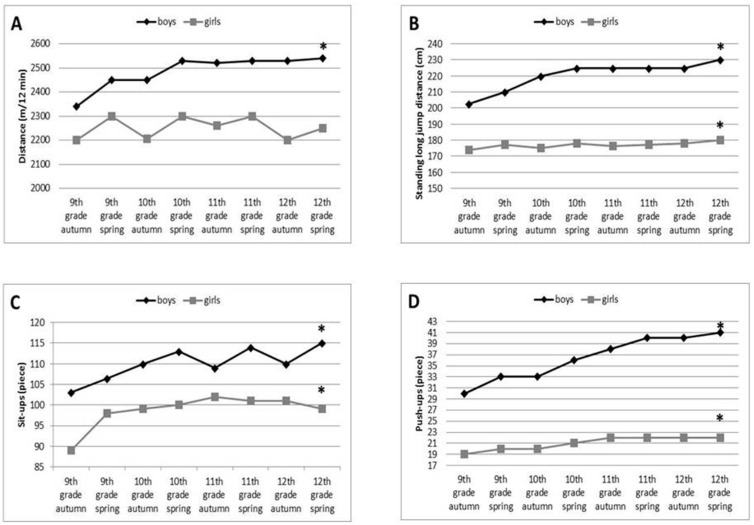
Changes in muscle strength and aerobic fitness between 9th and 12th grade in both genders. Significant improvement was detected in boys’ aerobic capacity according to 9th grade autumn and 12th grade spring data (data are shown as median, * = *p* < 0.05). However, only a seasonal variation was revealed in girls’ aerobic capacity during the 4 years (**A**). Significant improvement was detected in leg dynamic muscle strength (measured with long jump distance) (**B**), in the strength and endurance of abdominal muscles (measured by sit-up tests) (**C**), shoulder and arm muscles (measured by push-up tests) (**D**) in both genders during the 4-year observational period (data are shown as median, * = *p* < 0.05).

There was a statistically significant difference between the 4-year run–walk test results of genders (girls: χ^2^(2) = 52.32, *p* < 0.001; boys: χ^2^(2) = 93.64, *p* < 0.001). There were significant differences between the 9th grade autumn and 12th grade spring run-walk test results among boys (Z = −4.726, *p* < 0.001, η^2^ = 11.99) (Figure 1A). Among girls, only a seasonal variation could be observed (there was no significant difference between the 9th grade autumn and 12th grade autumn data in girls; Z = −0.569, *p* = 0.569, η^2^ = 12.01).

#### 3.1.2. Muscle Performances of Boys and Girls during the Four Years

The lower limb dynamic, the strength and endurance of hip flexors and abdominal muscles, shoulder and arm muscle strength, significantly improved during the four years in both girls {1: χ^2^(2) = 67.147, *p* < 0.001; 2: χ^2^(2) = 183.16, *p* < 0.001; 3: χ^2^(2) = 148, *p* < 0.001} and boys {1: χ^2^(2) = 336.395, *p* < 0.001; 2: χ^2^(2) = 73.169, *p* < 0.001; 3: χ^2^(2) = 210.542, *p* < 0.001}. There were significant differences between the 9th grade autumn and 12th grade spring results of the standing long jump distance (boys Z = −10.404, *p* < 0.001, η^2^ = 11.95; girls Z = −4.153, *p* < 0.001, η^2^ = 12.004) (Figure 1B), sit-up test (boys Z = −3.269, *p* < 0.001, η^2^ = 12.003; girls Z = −8.073, *p* < 0.001, η^2^ = 11.98) (Figure 1C), and push-up test results (boys Z = −6.946, *p* < 0.001, η^2^ = 11.98; girls Z = −5.746, *p* < 0.001, η^2^ = 11.99) (Figure 1D).

### 3.2. Results from between-Weight-Groups Analyses

Association between obesity and overweight and cardiorespiratory performance.

**Figure 2:**
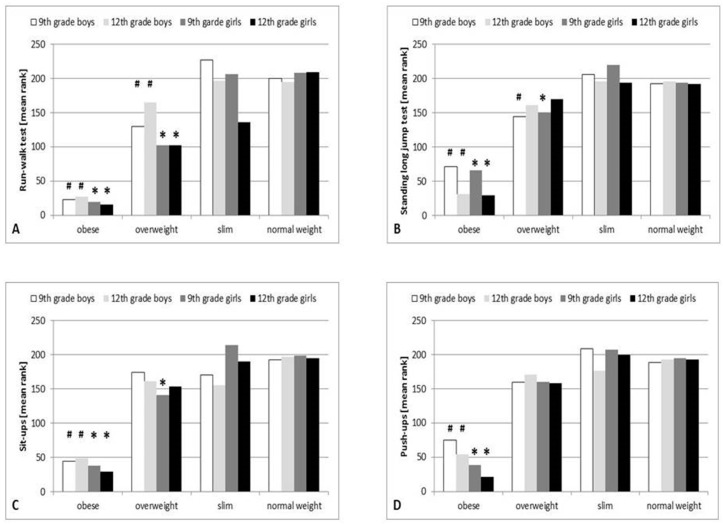
Association between body mass index (BMI)-for-age and aerobic and muscular fitness in adolescents. Run-walk test results are influenced by weight status in both genders (data are shown as mean rank, * = *p* < 0.05 for obese and overweight girls vs. normal weight girls; # = *p* < 0.05 for obese and overweight boys vs. normal weight boys) (**A**). Long jump test data were only influenced by obesity among 12th grade students (girls and boys, data are shown as mean rank, * = *p* < 0.05 for obese girls vs. normal weight girls; # = *p* < 0.05 for obese boys vs. normal weight boys) (**B**). Sit-up test results were significantly associated with obesity, performance and gender (data are shown as mean rank, * = *p* < 0.05 for obese girls vs. normal weight girls; # = *p* < 0.05 for obese boys vs. normal weight boys) (**C**). Push-up test results were significantly influenced by obesity, but not by overweight in both genders (data are shown as mean rank, * = *p* < 0.05 for obese girls vs. normal weight girls; # = *p* < 0.05 for obese boys vs. normal weight boys) (**D**).

The Kruskal–Wallis H test showed a significant association between weight status and run-walk test results: the performance of overweight and obese girls was significantly lower than that of classmates with normal weight in the 9th grade (χ^2^(2) = 102.943, *p* < 0.001, η^2^ = 0.292), and in the 12th grade (χ^2^(2) = 96.844, *p* < 0.001, η^2^ = 0.274). Similar results were observed among boys in 9th grade (χ^2^(2) = 109.655, *p* < 0.001, η^2^ = 0.33) and in 12th grade (χ^2^(2) = 86.406, *p* < 0.001, η^2^ = 0.258) (Figure 2A) (Table 1).

#### 3.2.1. Association between Obesity and Overweight and Lower Limb Performance

The performance of overweight and obese girls was significantly lower than that of normal weight girls in 9th grade (χ^2^(2) = 37.85, *p* < 0.001, η^2^ = 0.102). In 12th grade, only obese girls provided significantly lower performance χ^2^(2) = 44.341, *p* < 0.001, η^2^ = 0.121). Similar results were found among boys in 9th grade (χ^2^(2) = 50.906, *p* < 0.001, η^2^ = 0.148), and in 12th grade (χ^2^(2) = 82.886, *p* < 0.001, η^2^ = 0.247) (Figure 2B) (Table 1).

#### 3.2.2. Association between Obesity and Overweight and Hip Flexor and Abdominal Muscle Performance

Nonparametric analyses revealed an association between weight status and hip flexor and abdominal muscle strength in the adolescents. The median maximal performance of overweight and obese girls was significantly lower in 9th grade compared with normal weight girls (χ^2^(2) = 58.752, *p* < 0.001, η^2^ = 0.163). In 12th grade, only the obese girls’ performance was lower (χ^2^(2) = 47.980, *p* < 0.001, η^2^ = 0.132). Among boys, only obesity was associated with worsened results of the sit-up tests, but no association between overweight and abdominal muscle performance could be observed (χ^2^(2) = 65.282, *p* < 0.001, η^2^ = 0.193) neither in 9th nor in 12th grade (χ^2^(2) = 68.863, *p* < 0.001, η^2^ = 0.204) (Figure 2C) (Table 1).

#### 3.2.3. Association between Obesity and Overweight and Shoulder and Arm Muscle Performance

The push-up data were inversely associated only with obesity in girls (χ^2^(2) = 48.853, *p* < 0.001, η^2^ = 0.134 in 9th grade; χ^2^(2) = 51.102, *p* < 0.001, η^2^ = 0.141 in 12th grade) and similarly in boys (in 9th grade χ^2^(2) = 41.229, *p* < 0.001, η^2^ = 0.118; in 12th grade: χ^2^(2) = 57.717, *p* < 0.001, η^2^ = 0.169). The performance of overweight boys and girls was similar to the normal weight peers also in 9th and 12th grade (Figure 2D) (Table 1).

### 3.3. Regression Analyses

Multivariate linear regression and stepwise analyses of the data from baseline BMI, sit-up and cardiorespiratory performance in 11th grade were performed to predict cardiorespiratory performance at the end of the observational period. These variables statistically significantly predicted changes in end cardiorespiratory performance (F (5, 342) = 9752.34, *p* < 0.0005, R^2^ = 0.993).

## 4. Discussion

We have examined the association between the BMI-for-age and the cardiorespiratory and muscle performance of adolescents between 14 and 18 years of age. Our results show that cardiorespiratory performance increased significantly in boys during the 4 years, meanwhile in girls it only showed seasonal fluctuation. The strength of leg, shoulder and arm and abdominal muscles significantly increased both in boys and girls, but boys showed a more pronounced improvement. There was no significant change in the BMI-for-age during the examined period either in boys nor in girls. An inverse association between obesity and overweight and cardiorespiratory performance of the adolescents regardless of their age and gender was revealed. Worsening muscle performances were associated primarily with obesity. There was no association between overweight and shoulder and arm muscle performance in any age categories neither in girls nor in boys. Overweight was negatively correlated with lower limb strength in 9th grade girls and boys, and with abdominal muscle performance only in 9th grade girls.

The physical performance of adolescents in our study was similar to data of a recent survey examining children and adolescents in 30 European countries [36]. This study showed that boys performed better than girls in muscular strength, power and endurance and physical fitness generally improved at a faster rate in boys than in girls, especially during the teenage years. In our study it was also found that the cardiorespiratory performance of adolescent girls showed a seasonal fluctuation, as the results of spring surveys were always better compared to the preceding autumn results, and no continuous development could be observed. Although we have not examined the reason of this phenomenon it might be due to the lower physical activity and increased weight gain during the summer holiday among adolescent girls as was suggested in a previous research [37]. The strength and endurance of the lower limb, the abdominal, and the shoulder and arm muscles of both girls and boys significantly increased during the 4-year, but the improvement was significantly lower among girls, so the existing sex-differences further increased during this period (Figure 1B–D), which is also in accordance with previous data [36].

No significant change in the BMI-for-age was observed in our study neither in boys nor in girls during the 4-year. The proportion of normal weight girls remained the same (75%) and a slight decrease in the proportion of overweight (15% vs. 12%) and obese (6% vs. 5%) girls could be observed. With respect to the boys, a slight increase in the proportion of normal weight (69% vs. 72%), and a slight decrease in overweight (15% vs. 11%) boys could be measured, while the number of obese boys remained the same (10%) and was higher compared to that of girls’. In our sample of adolescents, the prevalence of obesity and overweight was lower than those reported in the literature [1,2].

The cardiorespiratory performance showed an inverse correlation with the BMI-for-age of the adolescents. The running distances of both overweight and obese girls and boys were significantly shorter than those of normal weight children (Figure 2A), regardless of their age. The association between the weight status and cardiorespiratory and muscle performances has been examined previously among adolescents. All studies concluded that there was a strong and inverse association between BMI status and cardiorespiratory performance [30,31,38,39]. However, the association between BMI and the muscle performance of adolescents shows greater variability. In a Finnish study it was found that both overweight and obesity significantly affected all aerobic and muscle tests (sit-ups, five-jump, back-and-forth jumping, ball skills, coordination, and endurance shuttle run tests), with the exception of the sit-and-reach test, and overweight and obesity had the most negative association with cardiorespiratory performance and muscle endurance tests [38]. Examining 9 different muscular strength tests and the body weights of Spanish children (1513 boys and 1265 girls) aged 6 to 17.9 years it was found that normal weight children showed significantly higher performance than their overweight and obese counterparts in lower body explosive strength tests and in the push- up test in boys and bent arm hang test in both boys and girls, and boys had significantly better scores than girls in all the studied tests [40]. Taiwanese youth aged 10 to 18 years showed decreased fitness levels for lower body explosive strength and cardiorespiratory endurance in both sexes for the obese and overweight children and adolescents (sit-up, flexibility and abdominal muscular strength/endurance) [39].

In our study, obesity showed negative association with the performance of all examined muscle groups (lower limb, shoulder and arm and abdominal), and this inverse association could be observed throughout the 4 years and no gender differences could be observed. However overweight had no such clear inverse association with the muscle performance of the adolescents. Only the lower limb muscle strength in 9th grade in boys and girls and the abdominal muscle strength and endurance in 9th grade girls were inversely associated with overweight. The shoulder and arm muscle performance were not affected by overweight neither in boys nor in girls in any age categories.

There are numerous studies in the literature to show that childhood obesity is strongly associated with increased risk of many chronic diseases in the short- and long-term [7,8,9,10,11,12,13]. Additionally, cardiorespiratory and muscle performance is independently and inversely associated with cardiovascular (CV) and metabolic risk factors in adolescents [7,9,25,26]. The Aerobics Center Longitudinal Study [41] and a Finnish cohort [42] also confirmed that better CV health in childhood promotes CV health in adulthood. Finally, we want to emphasize that physically active life in adolescence is associated with healthy weight, better cardio-metabolic health, increased bone mass, muscle strength and flexibility, improved academic performance, mental health, mood and sleep and social behavior [43].

### Limitations

Although we have obtained new data on the association of the BMI-for-age and physical performance of adolescents over a 4-year period, our study has also some limitations such as using only the BMI-for-age determining obesity and overweight, and body composition was not measured. Furthermore, the percentage of obese and overweight adolescents was relatively small in our sample, therefore further investigations are needed to establish our findings.

## 5. Conclusions

In our study the cardiorespiratory performance of adolescent girls and boys was inversely associated with both overweight and obesity, and this association could be observed throughout the 4 years. In addition, obesity also showed an inverse relation with muscle performance regardless of the age and gender of the adolescents. In contrast, the association between overweight and the strength of different muscle groups showed age and gender differences. Based on our data more attention is needed to maintain the normal weight status and physical performance of adolescents since increased BMI is negatively associated with most physical fitness parameters.

## Figures and Tables

**Table 1:** Mean rank values by the Kruskal–Wallis test for between weight-group analyses.

**GIRLS**						
**Performance Type**	**9th Grade**			**12th Grade**		
	**Obese**	**Overweight**	**Normal**	**Obese**	**Overweight**	**Normal**
run-walk	19.61	102.06	207.97	15.21	102.37	208.8
lower limb	65.77	150.18	193.78	28.66	169.88	191.42
hip flexor and abdominal muscle	37.45	140.99	198.26	29.21	153.57	194.29
shoulder and arm muscle	39.09	159.69	194.77	21.71	158.38	193.1
**BOYS**						
**Performance Type**	**9th Grade**			**12th Grade**		
	**Obese**	**Overweight**	**Normal**	**Obese**	**Overweight**	**Normal**
run-walk	22.97	129.59	200.5	27.37	165.21	195.11
lower limb	70.74	143.93	192.39	35.53	160.86	195.38
hip flexor and abdominal muscle	44.19	174.15	192.62	48.09	161.28	196.98
shoulder and arm muscle	75.28	159.66	188.1	54.29	159.66	192.52

## Data Availability

The data presented in this study are available on request from the corresponding author.
